# National Case Volumes and Gender Disparities in Emergency Department Utilization for Psychiatric Emergencies: A Population-Based Claims Data Analysis

**DOI:** 10.7759/cureus.66502

**Published:** 2024-08-09

**Authors:** Nnenna Okafor, Esther Okoro, Michael M Bojerenu, Nnaedozie Umeani, Daniel C Udegbe, Chinyere K Omeh, Chuka G Nwume, Tolulope D Alabi, Ishola A Fouhad, Victory Okpujie, Franklin A Andibanbang, Fidelis E Uwumiro

**Affiliations:** 1 Psychiatry, All Saints University College of Medicine, Kingstown, VCT; 2 Psychiatry, Cumbria, Northumberland, Tyne and Wear NHS Foundation Trust, Newcastle upon Tyne, GBR; 3 Psychiatry, St. Barnabas Hospital (SBH Health System), New York, USA; 4 Psychiatry, College of Medicine, University of Lagos, Lagos, NGA; 5 Psychiatry, Godfrey Okoye University Teaching Hospital, Enugu, NGA; 6 Psychiatry, 161 Nigerian Air Force Hospital, Makurdi, NGA; 7 Family Medicine, University of Port Harcourt, Port Harcourt, NGA; 8 Psychiatry, Priory Hospital, Roehampton, GBR; 9 Psychiatry, College of Medicine, Lagos State University, Lagos, NGA; 10 Internal Medicine, Central Hospital Benin, Benin City, NGA; 11 Psychiatry, Priory Hospital Ticehurst House, East Sussex, GBR; 12 Internal Medicine, Southern Regional Medical Center, Riverdale, USA

**Keywords:** catatonic syndrome, panic attacks, eating disorder, anxiety disorder, self-harm, abuse, suicidal ideation, bipolar disorder, depression, psychiatric emergencies

## Abstract

Introduction

The utilization of emergency departments (EDs) for managing psychiatric emergencies has significantly increased in the United States because of the increasing prevalence of mental health disorders. This study examined national case volumes and sex disparities in ED visits for psychiatric emergencies using data from the Nationwide Emergency Department Sample (NEDS).

Methods

This retrospective analysis included adult ED visits for psychiatric emergencies identified using relevant International Classification of Diseases, 10th Revision (ICD-10) codes. Primary endpoints included national case volumes by sex. Hospitalizations with age < 18 years and those with missing data on sex were excluded. Secondary endpoints included inpatient mortality, ED and inpatient costs, admission rates, discharge disposition, length of stay (LOS), and number of procedures.

Results

In 2021, there were approximately 143.5 million ED visits in the United States, with 7,978,490 of these being for psychiatric emergencies. The most common presentations were substance abuse and intoxication (5,119,086 (64.2%)), severe bipolar disorder (1,912,670 (24%)), and anxiety or panic attacks (1,015,486 (12.7%)). Approximately 3,997,223 (50.1%) were women, and 3,981,267 (49.9%) were men. Men were older (mean age: 45 versus 43 years; P<0.001), were more likely to be uninsured (712,647 (17.9%) versus 497,658 (12.5%); P<0.001), and had a higher Charlson Comorbidity Index (CCI) (CCI ≥ 2: 792,272 (19.9%) versus 643,552 (16.1%); P<0.001). More men than women presented to the ED with acute substance abuse or intoxication (3,196,945 (80.3%) versus 1,922,142 (48.1%)), bipolar disorder with or without psychosis (958,275 (24.1%) versus 954,395 (23.9%); P<0.001), and suicidal ideation (267,638 (6.7%) versus 208,989 (5.2%); P<0.001). More women than men presented with severe depression (455,683 (11.4%) versus 441,921 (11.1%)), anxiety and panic attacks (615,572 (15.4%) versus 402,108 (10.1%)), acute stress reaction (35,975 (0.9%) versus 23,888 (0.6%)), eating disorders (3,997 (0.1%) versus 27,869 (0.07%)), and a history of abuse (21,164 (0.53%) versus 19,569 (0.49%); P<0.001). Women had lower mortality rates (27,980 (0.7%) versus 63,956 (1.6%); P<0.001), lower mean ED costs (adjusted mean difference (AMD): $1,189; P<0.001), fewer in-hospital admissions (1,211,158 (30.3%) versus 1,453,162 (36.5%); P<0.001), and a higher number of prolonged hospitalizations (1,442,998 (36.1%) versus 1,194,380 (30%); P<0.001) compared with men.

Conclusion

This study highlights significant sex disparities in ED utilization for psychiatric emergencies. Men more frequently present with substance abuse and severe comorbidities, leading to higher healthcare costs and inpatient admissions. Women, while more likely to present with anxiety and depressive disorders, incur lower costs and have better overall outcomes.

## Introduction

The role of emergency departments (EDs) as the initial contact point for healthcare has become an important health system metric, being the only point of access for certain patients. In the United States, mental illness significantly affects well-being, with current data indicating that one in five US adults have a mental illness and approximately one in 25 adults have a severe mental illness, many of whom do not seek medical care [1,2]. Recently, US emergency departments (EDs) have experienced an increase in psychiatric cases, offering an initial point of contact for distressed individuals. Consequently, the use of EDs as the first point of contact has become a key health system performance measure, especially because the ED may be the sole access point for some patients [3,4].

Sex disparities in ED use continue to be a pressing challenge, particularly in the context of psychiatric care, where patterns of emergency presentations may differ by sex. Research has demonstrated distinct patterns in the prevalence and presentation of mental health conditions among men and women, which impact their use of ED services [5-7]. For instance, women are more likely to be diagnosed with mood and anxiety disorders, whereas men are more frequently diagnosed with substance use disorders and antisocial behaviors [8,9]. Since the COVID-19 pandemic, studies have shown that although the overall ED volume declined between 2019 and 2020, the proportion of patients with mental health conditions increased [10]. Given the persistent rise in patients presenting with psychiatric emergencies, it is imperative to closely examine this trend because an increase in ED utilization for mental health disorders necessitates changes in resource allocation, including adjustments in staffing, training, and relationships with referral services in the ED. At present, limited research has explored sex disparities in the use of EDs for specific psychiatric emergencies. Therefore, comprehensive data on the prevalence, trends, demographic characteristics, and outcomes, such as hospital admission rates, length of stay (LOS), and mortality, among sexes in EDs across the nation are lacking.

The objectives of the current study are to assess national volumes and sex disparities in ED presentation, resource use, and outcomes among patients with psychiatric disorders. This extensive examination will expand upon the existing body of knowledge by investigating current trends and addressing queries that have yet to be explored, with the ultimate objective of optimizing the distribution of resources and enhancing patient care within emergency departments.

## Materials and methods

This retrospective study was based on the publicly available Nationwide Emergency Department Sample (NEDS) database, which was deemed exempt from Institutional Review Board (IRB) approval due to its use of public de-identified data. We queried the Healthcare Costs and Utilization Project (HCUP) NEDS for all ED visits for psychiatric emergencies in 2021. The NEDS is the largest all-payer ED database in the United States, providing national estimates of hospital-owned ED visits. In its unweighted form, the NEDS included data from approximately 30 million ED visits in 2021. After weighting, it estimated 140 million ED visits. This study included discharge data for ED visits from 993 hospitals in 39 states and the District of Columbia, representing a 20% stratified sample of US hospital-owned EDs. The dataset encompasses demographic information such as hospital teaching status, patient age, race/ethnicity, geographic details such as hospital region, and the nature of ED visits, including primary reasons for ED visits (primary diagnoses) and all other diagnoses, including comorbidities and complications (secondary diagnoses). Additionally, it offers ED resource use information, such as the cost of ED services and inpatient care for 95% of visits, regardless of the expected payer, and includes data from children's hospitals with trauma centers, classified alongside adult and pediatric trauma centers in the current versions of the NEDS. Additional information on the NEDS can be found at https://hcup-us.ahrq.gov/nedsoverview.jsp1 and in previous studies based on NEDS data [11-13]. The primary endpoints of this study were nationally weighted estimates of ED presentations for psychiatric emergencies in men and women. The secondary analyses calculated inpatient mortality, cost of ED and inpatient services, rates of inpatient admissions, non-home discharge, length of hospital stay, prolonged hospitalizations, and number of procedures.

Cohort selection and study variables

We first queried the NEDS dataset for all adult ED visits for mental diseases and disorders using the major diagnostic category (MDC-19). Consistent with previously validated methodology [14,15], we identified ED presentations for which acute disorders of thought, behavior, mood, or social relationships requiring immediate intervention were the primary reasons for the ED visit (primary diagnosis). Using relevant International Classification of Diseases, 10th Revision (ICD-10) codes, we included all ED visits for suicidal ideation (R45.851), homicidal ideation (R45.850), non-suicidal self-harm (R45.88, X71-X79, and X78-X83), severe depression (F32), alcohol and drug withdrawal syndromes (F10, F11, F12, F14, and F19), acute exacerbations of bipolar disorder (mixed, depressed, manic, or hypomanic episodes with or without psychosis) (F31), mania (F30 and F31), catatonic/dystonic syndromes (F061, F20, and G24), anxiety, panic disorder/panic attacks (F41 and F40), dissociative identity disorder, dissociative amnesia, or depersonalization-derealization syndrome (F44 and F48.1), acute psychotic episodes (F20, F22, F23, F24, and F25), acute stress reactions (F43), eating disorders (F50), acute post-traumatic stress disorder (PTSD), abuse (physical, financial, psychological, and sexual), neglect, and abandonment (Z62, T74, and T76), restlessness and agitation (R451), and other medication-related emergencies, including neuroleptic malignant syndrome and serotonin syndrome (G210 and T43225A). In-hospital mortality was defined in the NEDS cohort using the binary variable "died_edvisit." Resource utilization, including length of hospital stay, total ED and inpatient costs, and number of procedures, was defined using length of stay (LOS), total charge (THC), and the number of Current Procedural Terminology and Healthcare Common Procedure Codes (CPT/HCPCS) recorded for each hospitalization. Prolonged hospitalization was defined as the length of hospital stay in the 99th percentile for all stays within the specific subtype of a psychiatric emergency.

We also identified other relevant patient- and hospital-level variables, including age, insurance status (Medicare, Medicaid, private insurance, and self-pay), weekend versus weekday admissions, hospital region, control, teaching status, trauma level, and urban-rural designation. Discharge disposition from the ED included routine home discharges, transfers to short-term hospitals, other transfers, including skilled nursing facilities, intermediate care, and other types of facilities, home healthcare, discharges against medical advice, admissions to inpatient care, and discharges/transfers to court/law enforcement. Non-home discharge was defined as any other discharge from the ED other than routine home discharge.

Statistical analysis

National (weighted) estimates of 2021 ED case volumes for psychiatric emergencies were calculated using discharge weights as instructed by the HCUP website, with HCUP-recommended statistical techniques employed to calculate estimates and standard errors [16]. Accordingly, specific adjustments were made for weighting (discwt), stratification (neds_stratum), and clustering (hosp_ed), and subsequent analysis was based on the weighted sample. We performed basic descriptive statistics, including Chi-square tests, Student's t-tests, and Fisher's exact tests, to compare psychiatric emergencies between men and women. The normality of data distribution was assessed using the Shapiro-Wilk test. Numerical data were presented as mean±standard deviation (SD) for normally distributed data and as median±interquartile range (IQR) for non-normally distributed data. Nominal data are presented as absolute counts with accompanying percentages. Multivariate hierarchical logistic regression (mixed-effects) models with random intercepts for centers were used to compare study outcomes for each psychiatric emergency between male and female patients. The covariates for the multivariate logistic regression models were selected based on the univariate analysis of patient- and hospital-level variables using a threshold of α<0.10. To obtain more statistically stable multivariate regression models, we excluded multicollinear explanatory variables by estimating the variance inflation factor (VIF) and variance decomposition proportions (VDPs). Covariates with VIF of >5 were excluded from the final multivariate analysis [17]. The results of multivariate analyses are presented as adjusted odds ratios (aOR) for dichotomous outcomes or adjusted mean differences (AMDs) for costs and charges with a 95% confidence interval (CI). Negative adjusted mean values represent adjusted mean reductions in cost estimates. All statistical analyses were two-tailed, with a significance level set at α<0.005. Analyses were performed using Stata 18MP statistical software (StataCorp LLC, College Station, TX).

## Results

National case volumes

There was a total of 143,453,564 ED visits across the United States in 2021. A total of 2,011,615 ED visits for psychiatric emergencies were identified using our cohort selection criteria in the NEDS dataset (1.4% of all ED visits in the United States in 2021). When discharge and trend weights were applied to calculate the national estimates, 7,978,490 ED visits for psychiatric emergencies were identified across the United States in 2021 (Table 1). The most common ED presentations were drug abuse or intoxication (5,119,086 (64.2%)), severe bipolar disorder (1,912,669 (24%)), anxiety or panic attacks (1,015,486 (12.7%)), acute severe depressive episodes with suicidal ideation, psychotic symptoms, or functional impairment (896,514 (11.2%)), rapid metal deterioration, including delirium (657,230 (8.2%)), and suicidal ideation (476,627 (6%)) (Table 1).

**Table 1:** National Estimates of Emergency Department Visits for Psychiatric Emergencies Data is presented as total numbers with accompanying percentages (%). ^a^Withdrawal from opioids, cannabis, cocaine, or methamphetamines ^b^Includes bipolar disorder, mixed, depressed, manic, or hypomanic episodes with or without psychoses ^c^Depression with suicidal ideation or attempts, severe psychotic symptoms (hallucinations or delusions), frailty or functional impairment, or acute exacerbation of symptoms ^d^Neuroleptic malignant syndrome or serotonin syndrome ^e^Includes physical, psychological, or sexual abuse, and elder neglect, abuse, or abandonment ^f^Anorexia nervosa, bulimia nervosa, binge-eating disorder, pica, psychogenic vomiting, or avoidant/restrictive food intake disorder *Dissociative identity disorder, dissociative amnesia, or depersonalization-derealization syndrome

Psychiatric emergencies	Unweighted	Weighted
All psychiatric emergencies	2,609,485	7,978,490
Alcohol and drug abuse or withdrawal syndrome + delirium tremens^a^	1,301,642 (49.9)	5,119,086 (64.2)
Bipolar disorder^b^	476,084 (23.7)	1,912,669 (24)
Suicidal ideation	116,252 (5.8)	476,627 (6)
Non-suicidal self-harm	37 (0.002)	184 (0.002)
Homicidal ideation	4,958 (0.2)	20,365 (0.3)
Acute severe depressive episodes^c^	219,230 (10.9)	896,514 (11.2)
Acute manic episodes, unspecified	13,233 (0.7)	52,657 (0.7)
Rapid mental deterioration including delirium	161,475 (8)	657,230 (8.2)
Acute dystonic/catatonic syndrome	6,441 (0.3)	25,917 (0.3)
Anxiety/panic attacks ± agoraphobia	252,292 (12.5)	1,015,486 (12.7)
Dissociative disorders*	7,839 (0.4)	32,110 (0.4)
Agitation, unspecified cause	15,920 (0.8)	64,517 (0.8)
Medication-related emergencies^d^	458 (0.02)	1,834 (0.02)
Abuse^e^	10,486 (0.5)	40,732 (0.5)
Eating disorders^f^	1,942 (0.1)	7,979 (0.1)
Acute post-traumatic stress disorder	5,576 (0.3)	22,361 (0.3)
Acute stress reaction	15,620 (0.8)	61,520 (0.8)

Outcomes: Demographic characteristics and ED use

Out of 3,544,143 visits for psychiatric emergencies, approximately 50.1% (1,775,616) were women and 49.9% (1,768,527) were men. Men were older than women (45 years (IQR: 32-58 years) versus 43 years (IQR: 30-57 years); P<0.001), had fewer patients over 60 years old (788,291 (19.8%) versus 823,428 (20.6%); P<0.001), had a higher proportion of uninsured patients (712,647 (17.9%) versus 499,653 (12.5%); P<0.001), and had a higher comorbidity burden (Charlson Comorbidity Index (CCI) score ≥ 2: 792,273 (19.9%) versus 643,553 (16.1%); P<0.001). A higher proportion of men were admitted to inpatient care than women (1,453,162 (36.5%) versus 1,211,159 (30.3%); P<0.001). Men admitted to the ED for psychiatric emergencies had a higher prevalence of tobacco smoking (2,066,278 (51.9%) versus 1,734,795 (43.4%); P<0.001), hypertension (1,027,167 (25.8%) versus 931,353 (23.3%); P<0.001), hyperlipidemia (410,071 (10.3%) versus 343,761 (8.6%); P<0.001), congestive heart failure (274,707 (6.9%) versus 183,872 (4.6%); P<0.001), chronic renal disease (207,026 (5.2%) versus 151,894 (3.8%); P<0.001), and diabetes (366,277 (9.2%) versus 343,761 (8.6%); P<0.001). Women had a higher proportion of patients with obesity (143,900 (3.6%) versus 127,401 (3.2%); P<0.001), chronic obstructive pulmonary disease (COPD) (671,533 (16.8%) versus 565,340 (14.2%); P<0.001), and hypothyroidism (247,828 (6.2%) versus 91,569 (2.3%); P<0.001) (Table 2).

**Table 2:** Demographic Characteristics of Emergency Department Visits for Psychiatric Emergencies Stratified by Sex Categorical variables are compared using Chi-square tests and numerical variables using linear regression. P-values are considered significant at values <0.05. Data is presented as the total number with accompanying percentages (%) unless otherwise specified. LOS, length of hospital stay; US, United States; DRG, diagnosis-refined groups; CCI, Charlson Comorbidity Index; THC, total hospital charges; MI, myocardial infarction; CHF, congestive heart failure; COPD, chronic obstructive pulmonary disease; ED, emergency department; $US, United States dollar; ICD-10-PCS, International Classification of Diseases, 10th Revision, Procedure Coding System; CPT/HCPCS, Current Procedural Terminology/Healthcare Common Procedure Coding System; IQR, interquartile range

Variables	Women (N=3,997,223) (number (%) unless otherwise specified)	Men (N=3,981,267) (number (%) unless otherwise specified)	P
Median age (IQR) (years)	43 (30-57)	45 (32-58)	<0.001>
Age categories			<0.001>
18-40	1,834,725 (45.9)	1,680,095 (42.2)	
41-60	1,339,070 (33.5)	1,512,881 (38)	
61-80	639,556 (16)	696,722 (17.5)	
>80	183,872 (4.6)	91,569 (2.3)	
Insurance status			<0.001>
Medicare	1,099,236 (27.5)	1,007,261 (25.3)	
Medicaid	1,510,950 (37.8)	1,465,106 (36.8)	
Private	887,384 (22.2)	796,253 (20)	
Self-pay	499,653 (12.5)	712,647 (17.9)	
Discharge quarter			0.001
Q1 (January-March)	943,345 (23.6)	951,523 (23.9)	
Q2 (April-June)	1,019,292 (25.5)	1,015,223 (25.5)	
Q3 (July-September)	1,055,267 (26.4)	1,047,073 (26.3)	
Q4 (October-December)	975,322 (24.4)	963,467 (24.2)	
CCI			<0.001>
0	2,550,228 (63.8)	2,452,460 (61.6)	
1	803,442 (20.1)	740,516 (18.6)	
2	291,797 (7.3)	306,558 (7.7)	
≥3	351,756 (8.8)	485,715 (12.2)	
Type of ED event			<0.001>
ED visit in which the patient is treated and released	2,694,128 (67.4)	2,428,573 (61)	
ED visit in which the patient is admitted to this same hospital	1,211,159 (30.3)	1,453,162 (36.5)	
ED visit in which the patient is transferred to another short-term hospital	87,939 (2.2)	87,588 (2.2)	
ED visit in which the patient died in the ED	0 (0)	3,981 (0.1)	
ED visits in which the patient was not admitted, destination unknown	3,997 (0.1)	3,981 (0.1)	
ED visit in which the patient was discharged alive, destination unknown (but not admitted)	0 (0)	0 (0)	
Median household income national quartile for patient ZIP code			<0.001>
First (0-25th)	1,482,970 (37.1)	1,540,750 (38.7)	
Second (26th-50th)	1,087,245 (27.2)	1,062,998 (26.7)	
Third (51st-75th)	803,442 (20.1)	768,385 (19.3)	
Fourth (76th-100th)	623,567 (15.6)	609,134 (15.3)	
Hospital trauma center level			<0.001>
Non-trauma center	2,066,564 (51.7)	1,918,971 (48.2)	
Trauma level I	687,522 (17.2)	832,085 (20.9)	
Trauma level II	691,520 (17.3)	708,666 (17.8)	
Trauma level III	535,628 (13.4)	505,621 (12.7)	
Hospital teaching status			<0.001>
Metropolitan nonteaching hospital	875,392 (21.9)	800,235 (20.1)	
Metropolitan teaching hospital	2,594,198 (64.9)	2,727,168 (68.5)	
Nonmetropolitan hospital	527,633 (13.2)	453,864 (11.4)	
Hospital control/ownership			<0.001>
Government or private, collapsed category	555,614 (13.9)	545,434 (13.7)	
Government, nonfederal, and public	523,636 (13.1)	597,190 (15)	
Private, nonprofit, and voluntary	1,674,836 (41.9)	1,620,376 (40.7)	
Private, collapsed category	423,706 (10.6)	406,089 (10.2)	
Private, invest-own	819,431 (20.5)	812,178 (20.4)	
Hospital region (%)			<0.001>
Northeast	775,461 (19.4)	816,160 (20.5)	
Midwest	967,328 (24.2)	911,710 (22.9)	
South	1,411,020 (35.3)	1,365,575 (34.3)	
West	839,417 (21)	891,803 (22.4)	
Weekend admission	1,067,259 (26.7)	1,086,886 (27.3)	<0.001>
Patient location			<0.001>
"Central" counties of metro areas of ≥1 million populations	1,255,128 (31.4)	1,369,556 (34.4)	
"Fringe" counties of metro areas of ≥1 million populations	815,433 (20.4)	792,272 (19.9)	
Counties in metro areas with 250,000-999,999 people	859,403 (21.5)	836,066 (21)	
Counties in metro areas with 50,000-249,999 people	443,692 (11.1)	418,033 (10.5)	
Micropolitan counties	391,728 (9.8)	358,314 (9)	
Comorbidities			
Tobacco smoking	1,734,795 (43.4)	2,066,278 (51.9)	<0.001>
Hypertension	931,353 (23.3)	1,027,167 (25.8)	<0.001>
Obesity	143,900 (3.6)	127,401 (3.2)	<0.001>
Hyperlipidemia	343,761 (8.6)	410,071 (10.3)	<0.001>
CHF	183,872 (4.6)	274,707 (6.9)	<0.001>
Chronic renal disease	151,894 (3.8)	207,026 (5.2)	<0.001>
Uncomplicated diabetes types 1 and 2	343,761 (8.6)	366,277 (9.2)	<0.001>
Diabetes + complications	123,914 (3.1)	159,250 (4)	<0.001>
COPD	671,533 (16.8)	565,340 (14.2)	<0.001>
Old stroke	67,953 (1.7)	91,569 (2.3)	<0.001>
Hyperthyroidism	15,989 (0.4)	7,962 (0.2)	<0.001>
Hypothyroidism	247,828 (6.2)	91,569 (2.3)	<0.001>

Outcomes: Psychiatric emergencies stratified by sex

**Figure 1:**
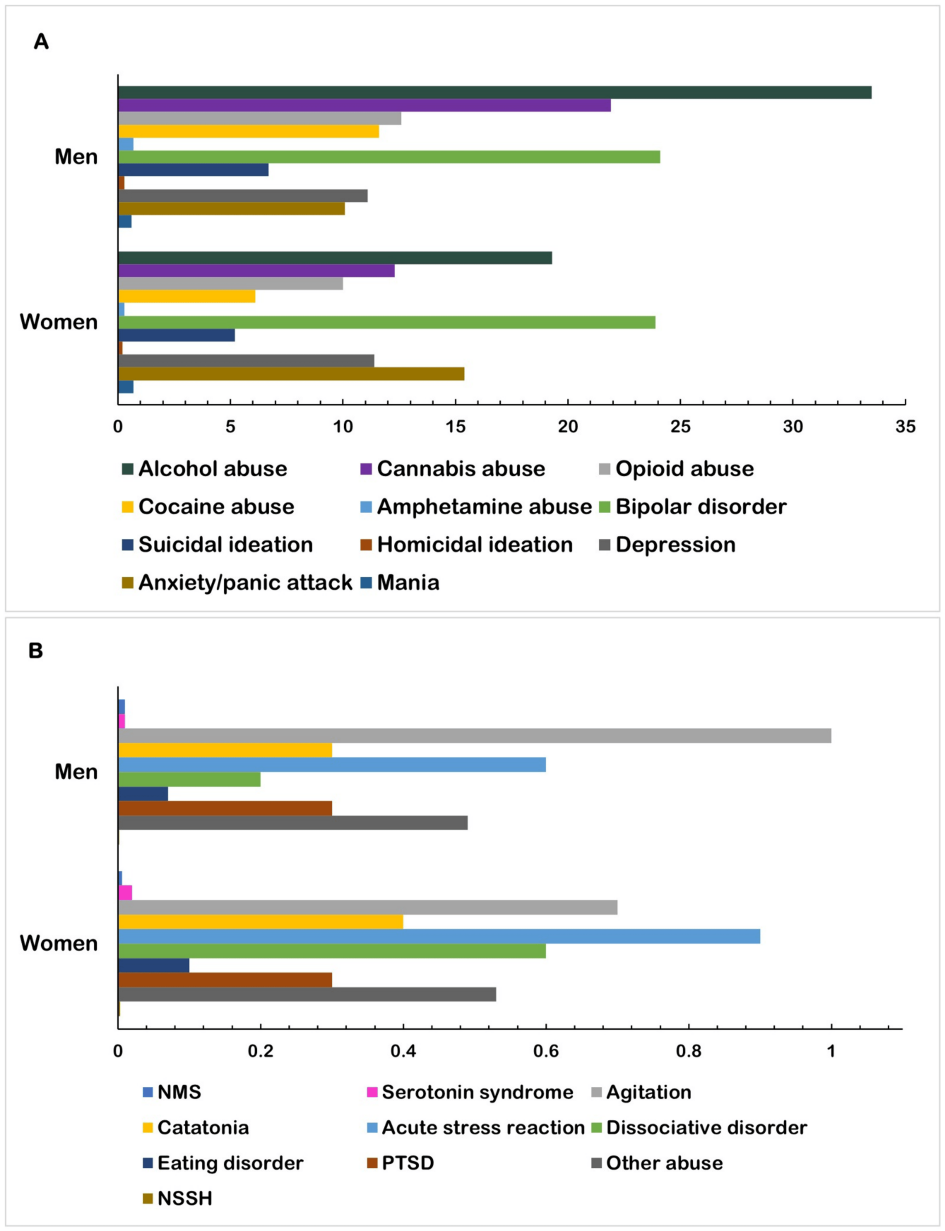
Prevalence of ED Visits for Psychiatric Emergencies by Sex NMS, neuroleptic malignant syndrome; PTSD, post-traumatic stress disorder; NSSH, non-suicidal self-harm; other abuse, physical, psychological, or sexual abuse, and elder neglect, abuse, or abandonment; ED: emergency department

Using the national estimates of ED visits for psychiatric emergencies, stratification was performed according to sex in the NEDS dataset (Table 3 and Figure 1). Although women accounted for a higher proportion of total ED visits for psychiatric emergencies (3,997,223 (50.1%) versus 3,981,267 (49.9%)), men had a greater proportion of any form of drug abuse, including alcohol (1,335,491 (33.5%) versus 773,143 (19.3%)), cannabis (871,448 (21.9%) versus 491,731 (12.3%)), opioid (500,504 (12.6%) versus 400,530 (10%)), cocaine (461,078 (11.6%) versus 243,501 (6.1%)), and amphetamine use disorder (28,424 (0.7%) versus 13,237 (0.3%); all: P<0.001). Men also had a greater proportion of acute exacerbations of bipolar disorder (958,275 (24.1%) versus 954,395 (23.9%)), suicidal ideation (267,638 (6.7%) versus 208,989 (5.2%)), homicidal ideation (13,521 (0.3%) versus 6,844 (0.2%)), and severe unspecified agitation (37,918 (1%) versus 26,600 (0.7%); all: P<0.001) (Table 3).

**Table 3:** Psychiatric Emergencies Stratified by Sex Proportions of psychiatric emergencies are compared using Chi-square tests with statistical significance set at P-values < 0.05. Data is presented as total counts with accompanying percentages (%). ^a^Includes bipolar disorder, mixed, depressed, manic, or hypomanic episodes with or without psychoses ^b^Depression with suicidal ideation or attempts, severe psychotic symptoms (hallucinations or delusions), frailty or functional impairment, or acute exacerbation of symptoms ^c^Anorexia nervosa, bulimia nervosa, binge-eating disorder, pica, psychogenic vomiting, or avoidant/restrictive food intake disorder ^d^Includes physical, psychological, or sexual abuse, and elder neglect, abuse, or abandonment *Dissociative identity disorder, dissociative amnesia, or depersonalization-derealization syndrome

Psychiatric emergency	Men (N=3,981,267)	Women (N=3,997,223)	P
Alcohol or drug abuse, intoxication, or withdrawal + delirium tremens (N=5,119,086)			
Alcohol abuse	1,335,491 (33.5)	773,143 (19.3)	<0.001>
Cannabis abuse	871,448 (21.9)	491,731 (12.3)	<0.001>
Opioid abuse	500,504 (12.6)	400,530 (10)	<0.001>
Cocaine abuse	461,078 (11.6)	243,501 (6.1)	<0.001>
Amphetamine use	28,424 (0.7)	13,237 (0.3)	<0.001>
Bipolar disorder^a^	958,275 (24.1)	954,395 (23.9)	<0.001>
Suicidal ideation	267,638 (6.7)	208,989 (5.2)	<0.001>
Homicidal ideation	13,521 (0.3)	6,844 (0.2)	<0.001>
Depression^b^	442,699 (11.1)	453,815 (11.4)	<0.001>
Acute manic episodes, unspecified	24,283 (0.6)	28,374 (0.7)	<0.001>
Anxiety/panic attack	401,596 (10.1)	613,890 (15.4)	<0.001>
Medication-related disorders (N=1,834)			
Neuroleptic malignant syndrome	425 (0.01)	239 (0.006)	<0.001>
Serotonin syndrome	331 (0.01)	836 (0.02)	<0.001>
Agitation	37,918 (1)	26,600 (0.7)	<0.001>
Acute dystonic/catatonic syndrome	11,744 (0.3)	14,173 (0.4)	<0.001>
Acute stress reaction	24,862 (0.6)	36,658 (0.9)	<0.001>
Dissociative disorder^*^	9,747 (0.2)	22,363 (0.6)	<0.001>
Eating disorder^c^	2,718 (0.07)	5,261 (0.1)	<0.001>
Acute post-traumatic stress disorder	11,072 (0.3)	11,289 (0.3)	0.249
Other abuse^d^	19,569 (0.49)	21,164 (0.53)	<0.001
Non-suicidal self-harm	74 (0.002)	110 (0.003)	0.009

Men had a higher proportion of ED visits for acute exacerbations of severe depression (153,815 (11.4%) versus 442,699 (11.1%)), mania (28,374 (0.7%) versus 24,283 (0.6%)), anxiety or panic attacks (613,890 (15.4%) versus 401,596 (10.1%)), catatonia (14,173 (0.4%) versus 11,744 (0.3%)), acute stress reaction (36,658 (0.9%) versus 24,862 (0.6%)), dissociative disorders (22,363 (0.6%) versus 9,747 (0.2%)), eating disorders (5,261 (0.1%) versus 2,718 (0.07%)), and other forms of abuse, including physical, financial, psychological, or sexual abuse, and elder neglect, abuse, or abandonment (21,164 (0.5%) versus 19,569 (0.5%); all: P<0.001) (Table 3). Women also presented to the ED for non-suicidal self-harm events more frequently than men (110 versus 74; P=0.009).

Medication-related disorders were also compared between men and women. A greater proportion of men presented with neuroleptic malignant syndrome (425 (0.01%) versus 239 (0.006%); P<0.001) than women, whereas women had a greater proportion of ED visits for serotonin syndrome (836 (0.02%) versus 331 (0.01%); P<0.001). No significant difference was observed in the frequency of ED visits for acute post-traumatic stress disorder between men and women (11,072 (0.3%) versus 11,289 (0.3%); P=0.249).

Outcomes: Mortality, resource utilization, and inpatient admissions

In the total cohort of psychiatric emergencies, 62,714 (1.6%) men and 26,586 (0.7%) women died during hospital visits, with an overall mortality rate of 1.1% (89,300). Women presenting to the ED for any psychiatric emergency had a lower likelihood of mortality than men (aOR: 0.86; 95% CI: 0.81-0.91; P<0.001). Additionally, women incurred $1,189 less in adjusted mean ED costs and $5,189 less in inpatient service costs (P<0.001), and they were less likely to be admitted to inpatient care (1,211,159 (30.3%) versus 1,453,162 (36.5%); P<0.001). However, among those admitted, women were more likely to have prolonged hospitalization (1,442,998 (36.1%) versus 1,194,380 (30%); P<0.001). For bipolar disorder (manic or hypomanic episodes) with or without psychosis, women had higher ED costs and lower inpatient service costs and were less likely to be admitted to inpatient care or discharged to non-home locations compared with men (all: P<0.001). There were no significant sex-based differences in mortality outcomes or mean length of hospital stay among patients with bipolar disorder.

For ED presentations with suicidal ideation, women incurred higher ED costs (AMD: $127; P=0.002) and had a higher rate of non-home discharges (115,571 (55.3%) versus 143,454 (53.6%); P<0.001) compared to men. On average, women received one more procedure than men (P<0.001) (Table 4). Similar outcomes were observed for presentations of severe homicidal ideations.

**Table 4:** Mortality, Resource Utilization, and Discharge Disposition for ED Visits Stratified by Sex P-values are considered significant at <0.05. *Adjusted mean difference in $US (negative values indicate lower values for women compared to men; positive numbers indicate higher mean differences for women compared with men); adjusted for insurance status, length of hospital stay, Charlson Comorbidity Index, and number of procedures performed ^a^Adjusted for age, day of admission, discharge quarter, illness severity, risk of mortality, insurance status, patient location, median annual income quartile, hospital region, trauma level and teaching status, and comorbidity burden ^b^Any ED discharge other than routine home discharge, including transfers to short-term hospitals, skilled nursing facilities, intermediate care, home healthcare, discharge against medical advice, and transfers to court/law enforcement ^c^Hospital length of stay in the 99th percentile CPT, Current Procedural Terminology; HCPCS, Healthcare Common Procedure Coding System; ED, emergency department; $US, United States dollar; IQR, interquartile range; SD, standard deviation; aOR, adjusted odds ratio; CI, confidence interval

Outcomes	Male	Female	aOR^a^ (95% CI)	P
All psychiatric emergencies	N=3,981,267	N=3,997,223		
All-cause mortality, number (%)	62,714 (1.6)	26,586 (0.7)	0.86 (0.81-0.91)	<0.001>
Cost of ED services, mean±SD ($US)	5,125±7,184	4,882±6,651	-189* (48-131)	<0.001>
Cost of inpatient services, mean±SD ($US)	56,016±98,868	47,351±75,567	-5,189* (4,138-6,240)	<0.001>
In-hospital admission, number (%)	1,453,162 (36.5)	1,211,159 (30.3)	0.76 (0.74-0.78)	<0.001>
Non-home discharge^b^, number (%)	1,938,877 (48.7)	1,710,811 (42.8)	0.79 (0.77-0.81)	<0.001>
Prolonged hospital stay^c^, number (%)	1,194,380 (30)	1,442,998 (36.1)	1.30 (1.27-1.33)	<0.001>
Length of hospital stay, mean±SD (days)	5.8±8.4	5.8±8.1	-	0.073
Number of CPT/HCPCS procedures performed, median (IQR)	7 (2-12)	8 (2-12)	-	0.001
Bipolar disorder	N=958,275	N=954,395		
All-cause mortality, number (%)	1,917 (0.2)	1,909 (0.2)	0.80 (0.73-1.88)	0.452
Cost of ED services, mean±SD ($US)	4,726±6,828	4,952±7,265	206* (114-298)	<0.001>
Cost of inpatient services, mean±SD	52,149±92,583	48,508±73,351	-3,721* (2,440-5,002)	<0.001>
In-hospital admission, number (%)	289,399 (30.2)	266,276 (27.9)	0.86 (0.83-0.89)	<0.001>
Non-home discharge^b^, number (%)	390,976 (40.8)	342,628 (35.9)	0.77 (0.75-0.80)	<0.001>
Prolonged hospital stay^c^, number (%)	265,442 (27.7)	286,319 (30)	1.16 (1.12-1.21)	<0.001>
Length of hospital stay, mean±SD (days)	5.4±7.9	5.0±6.3	-	0.083
Number of CPT/HCPCS procedures performed, median (IQR)	6 (2-12)	7 (2-13)	-	>0.001
Suicidal ideation	N=267,638	N=208,989		
All-cause mortality, number (%)	22 (0.01)	26 (0.12)	1.87 (0.51-6.88)	0.348
Cost of ED services, mean±SD ($US)	4,562±3,789	4,509±3,740	127* (49-206)	0.002
Cost of inpatient services, mean±SD ($US)	23,930±44,496	19,833±35,139	-2,407* (-9,484 to 4,670)	0.504
In-hospital admission, number (%)	2,141 (0.8)	1,463 (0.7)		0.121
Non-home discharge^b^, number (%)	143,454 (53.6)	115,571 (55.3)	1.10 (1.05-1.16)	<0.001>
Prolonged hospital stay^c^, number (%)	265,497 (99.2)	207,526 (99.3)	1.14 (0.97-0.34)	0.120
Length of hospital stay, median (IQR) (days)	2 (1-5)	2 (1-5)	-	0.959
Number of CPT/HCPCS procedures performed, median (IQR)	7 (3-10)	8 (3-11)	-	<0.001>
Homicidal ideation	N=13,521	N=6,844		
Cost of ED services, mean±SD ($US)	4,303±3,421	4,627±3,449	379* (132-627)	0.003
Cost of inpatient services, mean±SD ($US)	27,859±17,853	22,401±33,113	-9,245 (-5,285 to -13,215)	<0.001>
In-hospital admission, number (%)	41 (0.3)	27 (0.4)	1.09 (0.51-2.32)	0.827
Non-home discharge^b^, number (%)	7,883 (58.3)	4,366 (63.8)	1.24 (1.07-1.43)	0.003
Length of hospital stay, mean±SD (days)	6±3	3±4.5	-	<0.001>
Number of CPT/HCPCS procedures performed, median (IQR)	7 (3-10)	8 (4-11)	-	<0.001>
Depression	N=442,699	N=453,815		
Cost of ED services, mean±SD ($US)	3,425±3,298	3,425±3,541	54* (-17 to 124)	0.135
Cost of inpatient services, mean±SD ($US)	21,525±36,115	22,091±28,274	1,248* (459-2,036)	0.002
In-hospital admission, number (%)	147,861 (33.4)	152,482 (33.6)	1.04 (0.99-1.08)	0.131
Non-home discharge^b^, number (%)	247,026 (55.8)	248,690 (54.8)	0.98 (0.94-1.02)	0.290
Prolonged hospital stay^c^, number (%)	296,608 (67)	301,787 (66.5)	0.97 (0.92-1.01)	0.150
Length of hospital stay, mean±SD (days)	6±6.2	8±6	-	0.001
Number of CPT/HCPCS procedures performed, median (IQR)	6 (2-9)	7 (2-10)	-	<0.001>
Acute manic episodes, unspecified	N=24,283	N=28,374		
Cost of ED services, mean±SD ($US)	3,783±3,750	3,746±3,504	-104* (-234 to 25)	0.113
Cost of inpatient services, mean±SD ($US)	37,071±58,061	37,230±46,744	1,271* (-3,453 to 912)	0.254
In-hospital admission, number (%)	12,311 (50.7)	14,726 (51.9)	1.04 (0.95-1.14)	0.384
Non-home discharge^b^, number (%)	18,746 (77.2)	19,014 (78.3)	1.05 (0.96-1.16)	0.276
Prolonged hospital stay^c^, number (%)	12,117 (49.9)	11,826 (48.7)	0.96 (0.88-1.05)	0.391
Length of hospital stay, mean±SD (days)	9.8±11.6	10±10.9	-	0.352
Number of CPT/HCPCS procedures performed, median (IQR)	8 (4-12)	9 (4-13)	-	0.001
Anxiety or panic disorders or attacks	N=401,596	N=613,890		
All-cause mortality, number (%)	30 (0.007)	0 (0)	1	-
Cost of ED services, mean±SD ($US)	2,981±3,261	3,224±3,588	206* (161-251)	<0.001>
Cost of inpatient services, mean±SD ($US)	22,871±21,207	25,966±24,359	1,191* (-115 to 2,496)	0.074
In-hospital admission, number (%)	6,585 (1.6)	10,383 (1.7)	0.94 (0.87-1.01)	0.090
Non-home discharge^b^, number (%)	27,309 (6.8)	37,447 (6.1)	0.86 (0.83-0.90)	<0.001>
Prolonged hospital stay^c^, number (%)	395,170 (98.4)	604,068 (98.4)	1.07 (0.99-1.16)	0.098
Length of hospital stay, mean±SD (days)	3.4±4	3±4	-	0.514
Number of CPT/HCPCS procedures performed, median (IQR)	3 (1-8)	4 (1-9)	-	<0.001>
Agitation or restlessness	N=37,918	N=26,600		
All-cause mortality, number (%)	9 (0.02)	0 (0)	1	-
Cost of ED services, mean±SD ($US)	3,797±4,821	3,995±5,464	666* (-114 to 248)	0.471
Cost of inpatient services, mean±SD ($US)	42,363±55,531	39,248±50,863	-4,990 (-7,081 to 7,101)	0.418
In-hospital admission, number (%)	607 (1.6)	346 (1.3)	0.73 (0.54-1.00)	0.050
Non-home discharge^b^, number (%)	8,873 (23.4)	6,783 (25.5)	1.04 (0.96-1.14)	0.320
Prolonged hospital stay^c^, number (%)	37,311 (98.4)	26,254 (98.7)	1.36 (0.97-1.86)	0.053
Length of hospital stay, mean±SD (days)	7.2±14.3	6.8±14	-	0.596
Number of CPT/HCPCS procedures performed, median (IQR)	6.7 (6.4)	7.4 (6.6)	-	0.002
Alcohol or drug abuse, intoxication, or withdrawal + delirium tremens	N=3,196,945	N=1,922,142		
All-cause mortality, number (%)	61,005 (1.9)	25,326 (1.3)	0.85 (0.81-0.89)	<0.001>
Cost of ED services, mean ±SD ($US)	5,522±7,880	5,394±7,576	-47* (-115 to 22)	0.180
Cost of inpatient services, mean±SD ($US)	58,708±103,107	51,201±83,338	-5,552 (-6,758 to -4,347)	<0.001>
In-hospital admission, number (%)	1,448,216 (45.3)	834,210 (43.4)	0.97 (0.94-1.00)	0.094
Non-home discharge^b^, number (%)	1,691,184 (52.9)	972,604 (50.6)	0.95 (0.92-0.98)	0.001
Prolonged hospital stay^c^, number (%)	1,761,517 (55.1)	1,095,621 (57)	1.02 (0.99-1.06)	0.148
Length of hospital stay, mean±SD (days)	5.5±7.7	5.2±6.8	-	0.064
Number of CPT/HCPCS procedures performed, median (IQR)	8 (3-14)	9 (3-15)	-	<0.001>
Acute dystonic/catatonic syndrome	N=11,744	N=14,173		
All-cause mortality, number (%)	19 (0.2)	41 (0.3)	1.02 (0.27-3.80)	0.978
Cost of ED services, mean±SD ($US)	3,743±5,009	4,171±12,719	306* (-338 to 1,077)	0.306
Cost of inpatient services, mean±SD ($US)	61,845±82,928	60,952±113,883	-1,874* (-13,095 to -9,347)	0.743
In-hospital admission, number (%)	1,879 (16)	1,970 (13.9)	0.79 (0.66-0.95)	0.011
Non-home discharge^b^, number (%)	3,030 (25.8)	3,005 (21.2)	0.69 (0.600-0.80)	<0.001>
Prolonged hospital stay^c^, number (%)	6,858 (54.8)	8,022 (56.6)	1.03 (0.99-1.06)	0.105
Length of hospital stay, median (IQR) (days)	7 (3-14)	6 (3-12)	-	0.043
Number of CPT/HCPCS procedures performed, median (IQR)	4 (2-9)	4 (2-10)	-	0.496
Acute stress reaction	N=24,862	N=36,658		
Cost of ED services, mean±SD ($US)	3,080±3,503	3,268±3,442	98* (-31 to 229)	0.138
Cost of inpatient services, mean±SD ($US)	15,722±16,147	23,789±28,240	6,770* (3,628-9,911)	<0.001>
In-hospital admission, number (%)	1,492 (6)	2,053 (5.6)	0.92 (0.76-1.11)	0.362
Non-home discharge^b^, number (%)	2,983 (12)	3,666 (10)	0.82 (0.72-0.94)	0.003
Prolonged hospital stay^c^, number (%)	23,370 (94)	34,642 (94.5)	1.11 (0.91-1.35)	0.297
Length of hospital stay, mean±SD (days)	4±5	5±5.3	-	0.010
Number of CPT/HCPCS procedures performed, median (IQR)	3 (1-8)	4 (1-9)	-	<0.001>
Dissociative disorders	N=9,747	N=22,363		
All-cause mortality, number (%)	0 (0)	10 (0.04)	1 (omitted)	
Cost of ED services, mean±SD ($US)	5,482±7,179	5,329±7,049	-176 (-621 to 269)	0.438
Cost of inpatient services, mean±SD ($US)	44,377±44,552	39,578±34,558	-2,993 (-7,016 to 1,030)	0.145
In-hospital admission, number (%)	2,934 (30.1)	6,441 (28.8)	1.04 (0.92-1.19)	0.517
Non-home discharge^b^, number (%)	3,636 (37.3)	7,671 (34.3)	0.95 (0.84-1.07)	0.409
Prolonged hospital stay^c^, number (%)	6,862 (70.4)	16,012 (71.6)	0.94 (0.84-1.07)	0.416
Length of hospital stay, mean±SD (days)	4±9	3±4	-	0.130
Number of CPT/HCPCS procedures performed, median (IQR)	7 (2-12)	8 (3-13)	-	0.060
Eating disorders	N=2,718	N=5,261		
All-cause mortality, number (%)	0 (0)	6 (0.1)	1	-
Cost of ED services, mean±SD ($US)	3,085±3,100	3,429±3,982	249* (-255 to 754)	0.332
Cost of inpatient services, mean±SD ($US)	67,992±104,315	62,117±77,886	-7,558* (-30,787 to 15,669)	0.523
In-hospital admission, number (%)	658 (24.2)	1,957 (37.2)	2.01 (1.42-2.84)	<0.001>
Non-home discharge^b^, number (%)	872 (32.1)	2,336 (44.4)	1.78 (1.24-2.47)	0.001
Prolonged hospital stay^c^, number (%)	2,068 (76.1)	3,325 (63.2)	0.50 (0.35-0.71)	<0.001>
Length of hospital stay, mean±SD (days)	11±23	11±13	-	0.785
Number of CPT/HCPCS procedures performed, median (IQR)	4 (1-10)	8 (3-12)	2.2* (1-3)	<0.001>
Acute post-traumatic stress disorder	N=11,072	N=11,289		
Cost of ED services, mean±SD ($US)	3,252±3,764	3,443±6,172	311* (-37 to 660)	0.080
Cost of inpatient services, mean±SD ($US)	20,149±31,868	20,751±34,410	437 (-2,560 to 3,436)	0.774
In-hospital admission, number (%)	2,093 (18.9)	2,901 (25.7)	1.40 (1.15-1.69)	0.001
Non-home discharge^b^, number (%)	3,919 (35.4)	4,628 (41)	1.23 (1.05-1.44)	0.011
Prolonged hospital stay^c^, number (%)	9,057 (81.4)	8,410 (74.5)	0.72 (0.59-0.87)	0.001
Length of hospital stay, mean±SD (days)	5±10	6±10	-	0.944
Number of CPT/HCPCS procedures performed, median (IQR)	3 (1-8)	4 (1-10)	-	<0.001>
Abuse	N=19,569	N=21,164		
All-cause mortality, number (%)	0 (0)	16 (0.08)	1 (omitted)	
Cost of ED services, mean±SD ($US)	4,030±4,467	3,592±4,390	-567* (-48 to -1,086)	0.032
Cost of inpatient services, mean±SD ($US)	90,556±177,659	89,967±151,560	4,817* (-37,390 to 47,023)	0.823
In-hospital admission, number (%)	1,174 (6)	593 (2.8)	0.47 (0.33-0.68)	<0.001>
Non-home discharge^b^, number (%)	2,387 (12.2)	2,434 (11.5)	0.90 (0.67-1.21)	0.500
Prolonged hospital stay^c^, number (%)	18,414 (94.1)	20,571 (97.2)	2.13 (1.49-3.03)	<0.001>
Length of hospital stay, mean±SD (days)	12±21	12±30	-	0.954
Number of CPT/HCPCS procedures performed, median (IQR)	3 (1-7)	4 (1-8)	-	<0.001>
Medication-related disorders	N=50,418	N=41,484		
All-cause mortality, number (%)	706 (1.4)	249 (0.6)	0.18 (0.03-1.16)	0.071
Cost of ED services, mean±SD ($US)	3,438±3,974	2,774±2,712	-627* (-1,335 to 80)	0.082
Cost of inpatient services, mean±SD ($US)	104,273±170,365	92,618±132,669	-5,978* (-47,620 to 35,663)	0.778
In-hospital admission, number (%)	24,352 (48.3)	6,679 (16.1)	0.19 (0.11-0.36)	<0.001>
Non-home discharge^b^, number (%)	28,032 (55.6)	8,214 (19.8)	0.20 (0.12-0.35)	<0.001>
Prolonged hospital stay^c^, number (%)	26,469 (52.5)	34,805 (83.9)	4.9 (2.67-9.05)	<0.001>
Length of hospital stay, mean±SD (days)	10±11	9±8	-	0.810
Number of CPT/HCPCS procedures performed, median (IQR)	4 (1-9)	4 (2-9)	-	0.761

Mortality was not recorded in patients with acute depressive or manic states. However, women incurred higher inpatient costs (AMD: $1,248; 95% CI: $459-$2,036; P=0.002), longer hospital stays (8 versus 6 days; P<0.001), and a higher number of in-hospital procedures (7 versus 6; P<0.001). No significant sex disparities were observed in the cost of ED (P=0.113), inpatient services (P=0.254), in-hospital admissions (P=0.384), rates of non-home discharges (P=0.276), and prolonged hospital stay (P=0.391) (Table 4) for acute manic episodes. More women than men presented to the ED with acute severe anxiety or panic attacks/disorders (401,596 (10.1%) versus 613,890 (15.4%); P<0.001). Women incurred higher ED costs (AMD: $206; 95% CI: $161-$251; P<0.001) and had a higher mean number of in-hospital procedures (4 versus 3; P<0.001).

There were 64,518 ED admissions for unspecified agitation/restlessness, and the majority were men (37,918 (58.8%)) compared to women (26,600 (41.2%)). No significant sex disparities were observed in outcomes, including mortality, costs of ED and inpatient services, odds of in-hospital admissions, non-home discharge, length of hospital stay, or odds of prolonged hospital stay. ED visits for severe alcohol or drug intoxication were predominantly men (3,196,945 (62.5%)) compared with women (1,922,142 (37.5%)). Women had a lower likelihood of mortality (25,326 (1.3%) versus 26,005 (1.6%); aOR: 0.85; P<0.001), non-home discharge (972,604 (50.6%) versus 1,691,184 (52.9%); aOR: 0.95; P=0.001), and lower inpatient service costs (AMD: -$5,552; P<0.001) compared to men. On average, women received one more procedure than men (P<0.001). No significant differences were observed in ED costs, in-hospital admission rates, prolonged hospitalizations, and the mean length of hospital stay.

More women than men presented to the ED with acute dystonic or catatonic syndrome (14,173 (54.7%) versus 11,744 (45.3%)). Women patients had a lower likelihood of inpatient admission (1,970 (13.9%) versus 1,879 (16%); P=0.011) and a shorter mean hospital stay (6 days versus 7 days; P=0.043). Similarly, ED visits for acute stress reactions were predominantly women (36,658 (59.6%) versus 24,862 (40.4%)). Women had lower odds of non-home discharge (aOR: 0.82; P=0.003) and higher mean cost of inpatient care (AMD: $6,770; P<0.001). After adjustment, no significant sex disparities were observed in ED resource utilization, mortality, and length of stay for dissociative disorders.

There were 2,718 men and 5,261 women patients who presented to the ED with eating disorders. Women were more likely to be admitted to inpatient care from the ED (1,957 (37.2%) versus 658 (24.2%); P<0.001), less likely to have prolonged hospitalizations (3,325 (63.2%) versus 2,068 (76.1%); P<0.001), and received, on average, two more procedures than men (P<0.001).

## Discussion

The index study identified nearly eight million ED presentations for psychiatric emergencies across the United States in 2021. This high volume reflects the burden of psychiatric emergencies on ED resources. There were 164.38 million men and 167.51 million women in the United States in 2021 according to data from the US Census Bureau [18]. The current findings indicate that 2.38% and 2.42% of the total adult population (women and men, respectively) presented to the ED with at least one psychiatric complaint in 2021, a near-equal proportion of men and women. Complications of drug abuse or intoxication accounted for the majority of ED visits, followed by severe bipolar disorder, anxiety or panic attacks, and acute severe depressive episodes. Approximately 2.5 million ED visits were due to drug misuse, addiction, or abuse in 2011 [19]. Five million ED visits due to drug misuse, addiction, or abuse comprised 64.2% of the total ED visits in the index study. The increasing incidence of drug abuse is increasingly influencing mental health disorders among emergency department patients [20,21]. Emergency department (ED) visits for mixed, depressed, manic, or hypomanic episodes of bipolar disorder, with or without psychosis, accounted for up to 24% of the visits in this study. These cases were almost evenly split between genders, with 50.1% among men and 49.9% among women. Recent research has consistently shown a growing prevalence of bipolar disorder among women [22,23]. Our findings mirror this trend, indicating a near-equal prevalence of bipolar disorder episodes among men and women patients presenting to the ED.

Women have traditionally exhibited a higher likelihood of contemplating suicide, whereas men have a higher likelihood of dying by suicide [24], partly explaining why more men present to the ED for suicidal ideation or attempts. Shared risk factors for suicidal behavior in both genders included previous mental or substance use disorders and experiences of interpersonal violence. For women, specific risk factors for suicide include eating disorders, post-traumatic stress disorder, bipolar disorder, being a victim of dating violence, depressive symptoms, interpersonal issues, and a history of abortion. Women-specific risk factors for suicide include disruptive behavior or conduct issues, feelings of hopelessness, parental separation or divorce, suicidal behavior by friends, and access to other means. For men, risk factors for suicide death include drug abuse, externalizing disorders, and access to means for suicide completion [25,26].

Anxiety disorders have a lifetime prevalence of approximately 34% [27], whereas up to 4.7% of US adults have experienced panic attacks during their lifetimes [28]. In the index study, anxiety and panic disorders comprised 12.7% of ED visits for psychiatric emergencies, with a significant preponderance among women. The literature consistently shows that anxiety disorders occur more frequently in women than in men [29-31]. From a psychosocial perspective, masculinity is considered a protective factor for anxiety development, whereas femininity is a risk factor [32]. Studies that consider biological factors suggest that brain structures, genetic factors, and fluctuations in sex hormones may contribute to higher anxiety levels in women [33,34]. Biologically, women experience hormonal fluctuations, such as those associated with the menstrual cycle, pregnancy, and menopause, which can affect mood and anxiety levels. Brain structure differences and genetic predispositions also play a role [35]. Women also tend to have heightened sensitivity to stress, emotional stimuli, and trauma, such as abuse and domestic violence, which can contribute to the development of panic disorders. Social and cultural expectations, which often place greater emotional and caregiving burdens on women, can also increase their vulnerability to anxiety and panic. Although more women presented to the ED with anxiety/panic disorders and incurred higher ED costs, women were more likely to be discharged home from the ED and showed no difference in the likelihood of prolonged hospitalization compared with men.

Acute mental deterioration often represents an intercalation of medical and psychiatric causes and can result from a variety of factors, including severe infections (such as UTIs, pneumonia, and sepsis), adverse reactions to medications (particularly anticholinergics, sedatives, and pain medications), substance abuse and withdrawal, and metabolic imbalances, such as electrolyte disturbances and hypoglycemia. Neurological conditions such as strokes, transient ischemic attacks (TIAs), and brain tumors, as well as head trauma, dehydration, malnutrition, and hypoxia caused by respiratory or cardiac issues, are also common causes. In addition, psychiatric disorders (e.g., bipolar disorder, schizophrenia, and manic depression) and postoperative delirium, especially in older adults, contribute to sudden changes in mental status [36]. Other medication-related disorders (neuroleptic malignant syndrome and serotonin syndrome) were more prevalent among men, but women had a greater likelihood of prolonged hospitalization.

Consistent with the existing literature, the prevalence of eating disorders and abuse is higher in women [37,38] and has increased following the coronavirus pandemic [39]. Current evidence also links depression and anxiety disorders to a higher risk of eating disorders [40], both of which were more common in women in the present study. Our findings showed that women presenting to the ED with eating disorders were more likely to be hospitalized and undergo more procedures on average but less likely to have prolonged hospitalization. This study contributes to the existing literature on the prevalence and outcomes of eating disorders in the ED. Conversely, although more women than men sought ED care for abuse, men incurred higher ED costs and hospitalization rates. This may be related to the increasing prevalence of physical abuse among men with disabilities. The prevalence rates of physical violence against physically and mentally healthy men ranged from 3.4% to 20.3%, psychological violence from up to 37%, and sexual violence from 0.2% to 7%. For men with psychiatric disorders or disabilities, prevalence rates are significantly higher [41]. Although men with disabilities experience more physical violence, women with disabilities experience more sexual violence, humiliation, and discrimination [42].

Limitations

This nationwide claims data analysis has some limitations. The use of retrospective data from the NEDS restricted our ability to establish causality and relied on the accuracy of coding and documentation, which can introduce errors. The dataset lacks detailed clinical information, such as psychiatric condition severity, treatment specifics, and outcomes after hospitalization. Additionally, the NEDS excludes information on outpatient care or follow-up, which limits our understanding of long-term outcomes and readmissions. The data did not capture all social determinants of health that could influence the use of psychiatric emergency departments. Finally, the findings of this study may not be generalizable to non-hospital settings or populations not represented in the NEDS dataset.

## Conclusions

The findings of the index study reveal significant sex disparities. Men are more likely to present with substance abuse and severe comorbid conditions, resulting in higher healthcare costs and admission rates. In contrast, women more frequently present with anxiety and depressive disorders. Women incur lower costs and have better overall outcomes, including lower mortality rates. It is important to integrate screening for mental health disorders in both men and women into regular medical encounters and prioritize the development of early and targeted intervention strategies to address the specific needs of each patient, especially those with worsening psychiatric disorders. The current study also highlights the necessity for improved data collection and future prospective research to further understand the long-term outcomes and refine the management of psychiatric emergencies in the ED.
